# A Comparative Analysis of the Regulatory Framework and Collaborative Environment for Pediatric Medical Device Development in Japan and the United States: Identifying Challenges, Support Mechanisms, and Emerging Opportunities

**DOI:** 10.7759/cureus.68583

**Published:** 2024-09-03

**Authors:** Priyanka T R, Sudheer Kumar, Kamaraj R

**Affiliations:** 1 Pharmacy/M.Pharm Regulatory Affairs, SRM Institute of Science and Technology, Chennai, IND; 2 Pharmaceutical Regulatory Affairs, SRM Institute of Science and Technology, Chennai, IND; 3 Pharmacy, SRM Institute of Science and Technology, Chennai, IND

**Keywords:** regulatory measures, united states, japan, safety guidelines, regulatory compliance, medical device, pediatric

## Abstract

There are specific obstacles to designing medical devices for children, such as commercial distortions and regulatory hurdles. This study compares the regulatory frameworks and collaborative ecosystems of the United States (US) and Japan, enabling the development of pediatric medical devices. The study illustrates the differences as well as parallels between the two countries by outlining their primary obstacles, channels of assistance, and prospects.

The regulatory regimes of the US and Japan pose substantial challenges due to their intricate approval procedures and the dearth of pediatric-specific guidelines. However, while Japan's ecosystem is more dispersed, the US offers more well-established support mechanisms, such as funding initiatives and research alliances.

Despite these difficulties, there are still prospects for innovative thinking and collaborative work. The article demonstrates successful partnerships between business, academia, and government in both countries, which have helped propel the advancement of pediatric devices. For stakeholders endeavoring to maneuver through the complex terrain of pediatric medical device development, this study offers valuable insights. With a comprehensive understanding of the regulatory and collaborative frameworks in Japan and the US, developers can more effectively leverage resources, overcome obstacles, and bring vital technology to market. The study's findings have implications for researchers, industry leaders, and policy regulators, providing recommendations for strategies aimed at stimulating innovation and enhancing children's healthcare outcomes internationally.

## Introduction and background

The decreasing birthrate in Japan highlights the necessity for accelerated development and improvement of medical devices designed for pediatric care. However, companies face substantial obstacles when allocating resources to this field, including a constrained patient base, intricate disease manifestations, challenges in assessing device efficacy and safety, and the need for diverse device variants to address the heterogeneous needs of pediatric patients.

Classification of medical devices

In Japan, the Global Harmonization Task Force (GHTF) has established a classification system for medical devices, categorizing them into four risk-based classes. Classes I and II comprise low-risk devices that do not require in situ review criteria. In contrast, Classes III and IV consist of medium- and high-risk devices, respectively, which necessitate marketing approval from the Minister of Health, Labour, and Welfare (MHLW) before they can be marketed.

In Japan, the Pharmaceutical and Medical Devices Agency (PMDA) is responsible for reviewing the efficacy and safety of medical devices. These devices are categorized into three types: brand-new devices with a new configuration, improved devices that are existing products with enhancements, and me-too devices that are similar to already approved devices. In contrast, in the United States (US), the Center for Devices and Radiological Health (CDRH), a branch of the Food and Drug Administration (FDA), is tasked with reviewing medical devices to ensure their safety and effectiveness [1].

Age Definitions

The age threshold for adulthood differs significantly between Japan and the US, with Japan considering individuals 20 years of age or older as adults, whereas the US sets the threshold at 22 years of age or older [2]. This disparity has implications for the development and regulation of juvenile medical devices, highlighting the need for harmonized standards and definitions across countries.

Pediatric devices in the US are designed for patients under 21, creating a discrepancy. Treatment for congenital diseases often continues into adulthood, complicating age-based definitions. For the purposes of this study, pediatric medical devices refer to devices for underage patients and treatments for congenital diseases. To promote pediatric device development and regulatory approval, this study compares Japanese and US policies and proposes a strategy for Japan based on successful policies in both countries.

The pediatric medical device landscape is characterized by a significant innovation gap, wherein adult devices are frequently repurposed for pediatric use due to a dearth of devices specifically designed and marketed for children. This disparity stems from multifaceted barriers, including clinical challenges related to pediatric physiology, regulatory hurdles, and financial constraints [3].

Background

The Harmonization By Doing (HBD) program, inaugurated in 2003 as a trilateral collaboration between Japanese and American universities, enterprises, and government authorities, aimed to optimize global cardiovascular medical device development through process harmonization. However, recognizing the disparate pace of innovation in pediatric versus adult medical devices, HBD launched the HBD-for-Children initiative in 2016, specifically targeting the development of pediatric medical devices, thereby addressing the latent need for accelerated innovation in this underserved demographic [4].

## Review

Materials and methods

An examination of the PMDA website reveals a disproportionate approval rate of medical devices for pediatric populations, with only 12 (2.3%) of the 529 novel devices approved between 2006 and 2019 catering to pediatric or congenital conditions [5]. This study undertakes a comprehensive analysis of policies and sanctioned instances in Japan and the US, aggregating data from the PMDA, MHLW, FDA, and pertinent administrative websites, as well as scientific literature, to elucidate the regulatory landscape and support mechanisms for pediatric medical device development [2].

The HBD program adopts a comprehensive three-pronged approach to address the challenges in pediatric medical device development. Firstly, it engages with the industry through surveys to identify the obstacles hindering development. Secondly, it categorizes devices based on their prevalence worldwide and explores expedited appeal and licensing strategies in Japan and the US. Lastly, it facilitates transnational clinical assessments for pediatric medical devices in both countries, aiming to harmonize development and approval processes. By taking this multifaceted approach, the HBD program seeks to streamline and optimize the pathway for innovative devices to reach the market, ultimately addressing the disparities in pediatric medical device development [4].

Policies in Japan

As of December 2019, the MHLW and PMDA websites disclosed six key policies driving medical device development in Japan, with a focus on pediatric applications [6,7]. These policies comprise three initiatives from the MHLW: the Study Group on Early Introduction of Medical Devices [8], the Orphan Medical Device Designation System [9], and the SAKIGAKE Designation System [10]. Additionally, the PMDA has implemented three policies: the Conditional Early Approval System for Innovative Medical Devices Products [11], the PMDA Science Board on Evaluation of Medical Devices in Pediatric Use [12], and the Subsidization Program for Pediatric Medical Devices [13]. These policies collectively aim to accelerate medical device development in Japan, particularly for pediatric needs.

Our investigation delved into the objectives, accomplishments, and regulatory approval utilization of each policy, leveraging published data from the MHLW, PMDA, and JAAME, as well as company disclosures. To gain a deeper understanding of the "Orphan Medical Device Designation System," we supplemented our research with information from the Japan Association for the Advancement of Medical Equipment (JAAME) website, providing additional context and insights into this policy's role in promoting medical device development for rare diseases in Japan. This analysis provides insight into Japan's strategic framework for fostering medical device innovation, particularly in the pediatric sector.

Policies in US

Our investigation into US policies for pediatric medical devices employed a multifaceted approach. We conducted a PubMed search on August 11, 2019, using the search terms "Pediatric and Medical Device Regulation," which yielded 69 results. We then conducted an in-depth examination of four key papers [14] that summarized regulatory frameworks. Additionally, we performed a comprehensive review of FDA web pages, including "Pediatrics, Pediatric Medical Devices, and Public Meeting: Pediatric Medical Device Development," revealing nine initiatives that promote pediatric medical device development as of December 2019. By scrutinizing each policy's purpose and utilization status for pediatric medical device approval, using publicly available information on the FDA website, we gained a nuanced understanding of the US regulatory landscape for pediatric medical devices.

Outcomes

Efforts and Triumphs in Japan

In Japan, various initiatives have been implemented to promote the development of pediatric medical devices. These measures include the Subsidization Program for Pediatric Medical Devices, launched in 2013 [7], which aims to support the development of medical devices for children. Additionally, the Study Group on Early Introduction of Medical Devices with High Medical Need, established in 2013, has adopted 16 pediatric devices, accounting for 11.4% of the total [15]. The Orphan Medical Device Designation System, also introduced in 2013, has selected three pediatric devices, achieving a 100% approval rate [9]. Furthermore, the SAKIGAKE Designation System, launched in 2015, has selected one pediatric device, representing 1.1% of the total [10]. More recently, the Conditional Early Approval System for Innovative Medical Device Products was introduced in 2017 [11,16]. The objectives and consequences associated with each of these metrics are outlined in Table 1 [2].

**Table 1:** Japan’s encouraging regulatory regime for the invention of pediatric medical devices PMDA: Pharmaceuticals and medical devices agency

Name	Objective	Benefits for innovators	Inaugural year	Results
Study Group: Early Medical Device Introduction	Appraising the health significance of developments subsidized by business organizations that submit innovation requests and recognizing those who demonstrate an acute demand for healthcare in Japan	Prioritization analysis and reimbursement of incentives	2006	At the 29th conference in October 2018, 140 medical devices were officially established, and 16 (16/140, 11.4%) of them were juvenile medical devices. Furthermore, 7 (7/16, 43.8%) of the 16 pediatric devices were licensed
Designation System Concerning Orphan Medical Devices	Advocating for and supporting projects that establish orphan medical devices	Prioritization analysis, payment of grants, educating and conferring with regulatory agencies, favorable tax policies, and extension of the deadline for review	2013	As of December 2019, 30 medical supplies were officially designated, including 3 juvenile medical devices (10.0% of the total) that were approved following their declaration
PMDA Scientific Council on Medical Device Appraisal for Juvenile Use	PMDA advocates for the dissemination of medical breakthroughs, enhanced regulatory discipline, and rigorous screening of merchandise, leveraging modern technology and research to provide reliable and efficient drugs and medical devices	Considering factors, the review of the safety and efficient operation of medical devices for the pediatric population	2014	The PMDA Science Board issued a report headlined "Evaluation of Medical Device in Paediatric Use"
SAKIGAKE Scheme of Identification	Fostering research and development in Japan, with the goal of delivering cutting-edge medical devices to consumers promptly	Delivering personalized assistance, superior counsel, prioritization analysis, and reimbursement of incentives	2015	As of FY2019, nine medical devices had been categorized, with one (1%) being a juvenile device. Furthermore, an ongoing clinical trial is currently investigating these devices
System of Restricted Early Approval for Unique Medical Devices	Easing consumer access to the latest medical advances aimed at tackling serious illnesses for which there are currently no remedies, where the benefits outweigh potential hazards	Prioritization analysis, permitting manufacturers to capitalize on clinical trials conducted by the Cabinet Decree on Clinical Practice, and guidelines for medical devices within the framework of pre-market submissions with minimal clinical data	2017	In October 2018, the 29th meeting of the "Study Group on the Early Launch of Medical Devices" recommended endorsing a medical device for treating congenital cardiovascular disease under the legislation. However, as of the start of fiscal year 2019, this proposal remained unimplemented
Strategy to Grant Reimbursements for the Employing of Juvenile Medical Devices	Promoting and aiding the fabrication of juvenile medical devices	Payment of grants	2019	As of now, no medical device has been officially certified

Efforts and Triumphs in the US

Efforts and achievements aimed at streamlining the development of highly novel and orphan medical devices in the US are outlined in Table 2 [2]. To ensure access to innovative, reliable, and cost-effective medical products for orphan ailments and diseases affecting the pediatric population, the Office of Orphan Products Development and the Office of Pediatric Therapeutics were established. The Juvenile Report to Congress, published annually, reports on adopted pediatric medical devices, showing a consistent approval rate of 10%-20%. Furthermore, the FDA launched the Orphan Products Clinical Trials Grants Program in 1983 and the Pediatric Advisory Committee in 1999, issuing five guidelines for juvenile medical device development between 2003 and 2016 [17,18]. The Pediatric Medical Device Safety and Improvement Act, enacted in 2007 [19], promotes six key themes and initiatives, including oversight of adopted juvenile devices.

**Table 2:** United States' encouraging regulatory regime for the invention of pediatric medical devices FDA: Food and Drug Administration

Name	Objective	Benefits for innovators	Inaugural year	Results
Grants scheme for orphan products clinical investigations	Aiding in experimental investigations to create novel, secure, and effective pharmaceuticals for rare and peculiar illnesses and ailments	Payment of reimbursements	1983	At any given time, there are typically 60-85 operational grant-funded campaigns. Approximately $15.5 million of the annual budget is dedicated to fulfilling previously approved sponsorship obligations
Consultative panel on pediatrics	Directing and proposing juvenile-related research, clinical trials, pediatric branding, and adverse event monitoring to the Controller of Food and Drugs	No benefits	1999	The organization hosts annual gatherings, with three sessions having taken place in 2018
FDA regulations pertaining to the design of pediatric medical equipment	Relaying to enterprise and FDA professionals the FDA's current perspective on every concern	Taking into account the assessment of the reliability as well as efficiency of medical devices for the pediatric population	2003	Pediatric Competence for Advisory Panels (2003) Pediatric Medical Device Premarket Review (2014) Providing Insights on Medical Device Uses in Pediatrics (2014) Utilizing Relevant Clinical Data to Extrapolate Pediatric Medical Device Uses (2016)
Pediatric Medical Device Safety and Improvement Act (PMDSIA)	To improve the number of devices readily accessible to pediatric populations despite guaranteeing overall quality and safety, new financing sources of information, guidelines, enticements, and FDA's influence are being devised	Optimizing environment to facilitate the manufacture of pediatric medical devices	2007	The act spawned multiple initiatives designed to promote the fabrication of pediatric medical equipment
Humanitarian Use Device (HUD)/Humanitarian Device Exemption (HDE)	A marketing registration for a “humanitarian use device (HUD)” aimed to assist consumers with therapy or identification of an illness or condition which impacts or manifests	Payment of reimbursements sanctioned with anticipated perks	2008 (Revised HUD/HDE)	Out of the 418 certified medical devices, the FDA permitted 100 that were deemed appropriate to be employed in a pediatric population or demographic. Nine of the 100 (9/100, 9.0%) pediatric medical devices received FDA approval during the fiscal years 2009 and 2017 pursuant to "the Humanitarian Device Exemption (HDE) system"
Judiciary analysis	FDA fiscal year assessment of pediatric medical devices that have been authorized	Presenting details concerning juvenile medical devices that were officially certified	2008	The Pediatric Report to Congress comprised annually updated information on permitted juvenile medical devices
Pediatric Device Consortia (PDC) Grant Program	Aid in facilitating the creation of charity consortiums intended to inspire endeavors	Payment-grants	2009	During 2009, the campaign was awarded between $3.5 and $6 million annually
Breakthrough Device Program	Prioritizing and streamlining the fabrication of unique technologies for medicine enabling more precise assessment and management for fatal or persistently impairing ailments or diseases	Prioritization analysis expedited access route	2017 (2015)	As of September 30, 2018, the FDA had designated 97 medical devices as part of the Expedited Access Pathway Program or the "Breakthrough Device Program." As of the end of September 2018, nine (9/97, 9.3%) revolutionary devices had received approval for their promotional plan acknowledged, dismissed, or permitted
FDA Public Conference; Innovation of Pediatric Medical Devices	Exploring ideas for strengthening medical device ecology allows manufacturers to design and invent items that cater to children's exacerbated demands, thus hurrying up medical device technology for all Americans	Communicating perspectives between the regulatory body, industry, and academia	2018	The FDA hosted a public meeting in 2018 that addressed pediatric devices as an element of the "Breakthrough Devices medical device development"

The FDA has also restructured Humanitarian Use Device (HUD)/Humanitarian Device Exemption (HDE) approval pathway [20] and established the Pediatric Device Consortia (PDC) Grant Program, providing $3.5-6 million annually [21,22]. Furthermore, the Breakthrough Device Program was introduced in 2017, replacing the Expedited Access Pathway Program, with 97 selected devices and 24 approved investigational device exemptions (IDEs). These efforts aim to accelerate innovation and access to medical devices for orphan and pediatric populations. Japan's encouraging regulatory regime for the invention of pediatric medical devices is shown in Table 1 [2]. The US' encouraging regulatory regime for the invention of pediatric medical devices is shown in Table 2 [2]. Similar and contrast actions for pediatric medical device development in Japan and the US are shown in Tables 3-4 [2].

**Table 3:** Similar actions for pediatric medical device development in Japan and the US PMDA: Pharmaceuticals and medical devices agency; FDA: Food and Drug Administration

Similar actions	Main measures	Main measures
	Japan	United States
Reinvestigating medical devices for regulatory agencies	“PMDA Office of Medical Device I and II Assessment”	“Center for Devices and Radiological Health (CDRH) in FDA”
Suggestions to designers	Statement submitted by “PMDA Science Board”	“FDA guidance”
Grant program for the manufacturing of medical devices for juvenile	“Orphan Medical Device Designation System”	“Pediatric Device Consortia (PDC) Grant Program,” “Orphan Products Clinical Trials Grants Program”
Excluded from the requirement for approval	Mechanism of “Conditional Early Approval for Novel Medical Device Products”	“Humanitarian Use Device (HUD)/Humanitarian Device Exemption (HDE)”
Payment of reimbursements-filings and meetings with regulatory organizations	“Program for Orphan Medical Device Designation Program”	“HUD/HDE"
Preeminent assessment	Research Team on the Prompt Release of Medical Equipment and Other Items for High Medical Need The systems for designating orphan medical devices “SAKIGAKE designation system” “Conditional early approval for innovative medical device products”	“HUD/HDE,” “Breakthrough Device Program”
Prioritized consultation/accelerated access pathway	“SAKIGAKE Designation System”	“Breakthrough Device Program”

**Table 4:** Contrasts between the actions for pediatric medical device development in Japan and the United states FDA: Food and Drug Administration

Contrasts actions	Main measures	Main measures
	Japan	United States
“Specialized act for the legislative development of medical devices for children”	“Early Approval System with Constraints for Novel Medical Device Products” “SAKIGAKE Designation System”	“Pediatric Medical Device Safety and Improvement Act”
“FDA office which encourages medical devices for children”		“Office of Pediatric Therapeutics,” “Office of Orphan Products Development established in FDA”
“Autonomous pediatric medical device panel”		“Pediatric Advisory Committee”
“Specialized workgroup for the fabrication of pediatric medical devices”		“Pediatric Device Consortia (PDC) Grant Program”
“Gatherings for stakeholders from industry, academia and regulatory”		“FDA Public Meeting; Pediatric Medical Device Development”

In Japan, previous studies and reports have primarily focused on the number of pediatric devices accepted rather than the monetary amount of results. As a result, there is limited data available on the specific monetary amounts administered in Japan. Unlike in the US, where the monetary amounts of results are explicitly stated, Japanese reports have emphasized the quantity of approved pediatric devices, making it challenging to determine the corresponding monetary amounts.

Discussion

Comparative Analysis Between Japan and the US

A comparative examination of regulatory measures in Japan and the US (Tables 3, 4) reveals a temporal disparity, with the US implementing various steps primarily since the 2000s, whereas Japan's core criteria emerged in 2013. Despite this, both nations exhibit similarities in their approaches, including subsidies for R&D costs and enrollment fees, criteria for waivers, and the preeminent assessment and engagement of administrative authorities. However, Japan's lack of a "peculiar consortium" and limited budget for pediatric medical devices contrasts with the US. Nonetheless, the PMDA's priority review and consultation have been shown to accelerate the approval process for pediatric medical devices in Japan.

Effective Approach to Advance the Manufacturing of Medical Gadgets for Juveniles

Despite concurrent efforts in Japan and the US to promote pediatric medical device development, approvals have plateaued, necessitating a strategic reevaluation. Similar initiatives in both countries have yielded sporadic approvals, but stagnation persists. To revitalize development, a multifaceted approach is crucial, incorporating expedited regulatory review, global collaboration, and innovative evaluation methodologies. By addressing these key issues, Japan can revitalize pediatric medical device development and foster a more conducive environment for innovation.

To enhance pediatric medical device development, three key issues must be addressed: expediting regulatory review, supporting global collaboration, and implementing alternative evaluation methods. The FDA and PMDA have initiated preeminent analysis, leading to approvals. Global collaboration is crucial due to the limited number of target patients, and initiatives like the "Harmonization by Doing" (HBD) and "Collaboration Review" facilitate cooperation. Japan's "Conditional Early Approval System" [11] allows for early application with limited clinical data. Cooperation among stakeholders is essential for expediting approvals, and public meetings and programs, such as the HBD for children, can help identify and address barriers to innovation.

To overcome challenges in pediatric medical device development, two key strategies are proposed. Firstly, efficient clinical trial mechanisms, such as the support program offered by the Japanese Society of Pediatric Cardiology and Cardiac Surgery, can facilitate the development process. Secondly, nonclinical evaluation approaches, like those used for the EXCOR Pediatric Ventricular Assist Device and AMPLATZER Piccolo Occluder, can better assess device performance and safety for complex anatomies. These strategies can help address the limitations of small-scale clinical trials and ensure better effectiveness and reliability. Furthermore, insurance reimbursement remains a significant challenge; however, firms in Japan can leverage premium pricing divisions, such as the pediatric disease-specific medical devices and the Orphan Medical Device Designation System [9], to secure higher reimbursement rates. For instance, Abbott received a 20% extra expenditure for the AMPLATZER Piccolo Occluder. By utilizing regulatory policies, firms can also secure high insurance reimbursement, promoting pediatric medical device development.

The HBD program, launched in 2003, is a collaborative initiative between Japan and the US that aims to develop a multilateral system for leading-edge medical devices. By conducting proof-of-concept projects, HBD seeks to harmonize local regulations, enabling global clinical trials and simultaneous market approval. The program has yielded significant findings, including the similarity of essential principles in medical device regulation between Japan and the US; the efficiency and viability of harmonized clinical data collection for simultaneous applications; and the shared regulatory aspect of Good Clinical Practice (GCP) compliance. These insights have paved the way for streamlined global development and approval of innovative medical devices.

HBD for Children

The HBD for Children initiative was launched in 2016 as an international collaborative effort to promote the development of pediatric medical devices. This teamwork brings together regulatory agencies, industry leaders, and academic experts from Japan and the US to address the unique challenges in this field. The group's objectives include identifying flaws in the current system and proposing alternatives, streamlining pediatric medical device development, reducing research and financial burdens, establishing cost-effective pathways to market, simplifying global regulatory strategies, and enhancing safety through approved pediatric labeling. Additionally, the classification of Pediatric Medical Devices with high medical needs is outlined in Table 5 [4].

**Table 5:** Classification of pediatric medical devices with high medical needs

Category	Details
1	Approved in the US but not approved in Japan
2	Not approved but used as off-label in the US and Japan for a long time
3	Not approved and not used in the US and Japan but used/approved in other countries
4	Under development
5	Approved in Japan but not approved in the US

Recent Developments and Changes to Japan's Regulatory Framework for Pediatric Medical Devices

Japan is modernizing its regulatory framework for pediatric medical devices to align with global standards, notably with the MHLW revised guidelines for Software as a Medical Device (SaMD), issued in March 2023. These updated guidelines clarify regulatory distinctions between SaMD and non-SaMD, facilitating innovative healthcare software solutions for pediatric care. Additionally, the PMDA has streamlined its review systems to expedite approval for innovative medical devices, including those designed for pediatric use, as part of a comprehensive initiative to accelerate market entry for cutting-edge medical technologies. This aims to benefit children relying on advanced medical care by ensuring the swift development and deployment of safe, effective, and accessible pediatric medical devices in Japan.

Recent Developments and Changes to US Regulatory Framework for Pediatric Medical Devices

The US regulatory framework for pediatric medical devices has undergone significant developments, prioritizing safety, innovation, and timely access to essential devices for children. The FDA's PDC Grant Program continues to support innovators by funding consortia that provide regulatory, business, and clinical consultation services, thereby bridging the gap between device concept and market. Additionally, the FDA's Breakthrough Devices Program has been expanded to accelerate the development and review of life-saving pediatric devices, offering incentives such as prioritized review and increased interaction with the FDA. These updates aim to overcome the challenges associated with the smaller pediatric market and bring critical devices to market more quickly, ultimately improving the lives of children.

HDE Revisions

The FDA has made revisions to the HDE pathway, which allows for the approval of devices intended to treat or diagnose conditions affecting fewer than 8,000 individuals in the US annually. Given the rarity of many pediatric conditions, these changes are particularly relevant. The updates aim to streamline the process and encourage the development of devices that meet the unique needs of pediatric patients​.

Real-World Evidence (RWE) Integration

The FDA has been increasingly integrating RWE into the regulatory decision-making process for pediatric devices. RWE includes data collected from routine clinical practice, which can provide insights into the safety and effectiveness of devices post-approval. This shift allows for more flexible and responsive regulatory oversight, particularly important for pediatric devices where traditional clinical trials may be challenging to conduct​.

Pediatric Device Safety and Improvement Act

Recent legislative efforts, such as the PMDSIA, have sought to enhance the regulatory framework by requiring more rigorous post-market surveillance and safety monitoring for pediatric devices. This act also promotes the development of pediatric versions of adult devices, ensuring that children have access to appropriately sized and safe medical device.

Focus on Digital Health and AI

The FDA has been updating its guidelines and frameworks to better regulate pediatric devices that incorporate digital health technologies and artificial intelligence. These technologies offer new opportunities for pediatric care but also pose unique regulatory challenges, such as ensuring data privacy and algorithm transparency​. These developments reflect the FDA's commitment to improving the availability, safety, and effectiveness of pediatric medical devices, addressing both the unique challenges of pediatric care and the evolving technological landscape.

How are Pediatric Medical Devices Different?

Pediatric medical devices lag behind adult devices in terms of availability and technology, with only 25% of novel devices designed for pediatrics compared to adults. Most approved devices are for individuals aged 18 years or older, with few options for younger patients, leading to common off-label use of adult devices [23,24]. While off-label use is accepted, physicians agree that pediatric-specific solutions are needed. However, pediatric device development faces unique challenges in clinical, technical, regulatory, and financial domains. The major barriers to pediatric medical device development are shown in Table 6 [3].

**Table 6:** Major barriers to pediatric medical device development IRD: Institutional review board

Category	Barriers
Clinical considerations	Adult physiology is distinctive and evolves with time. Even for the same ailment, children may not benefit from the same diagnosis and path of treatment as adults. Typical daily routines for children might result in unanticipated challenges with device functionality
Technical considerations	Device engineering becomes problematic with diminutive dimensions and rising morphological advancement. Sustainability of devices additionally brings up issues with decay of materials, leaching, and biocompatibility
Regulatory and ethical considerations	Minimized sample size and populations that are diversified and dispersed throughout various institutions. Research on children who are terminally ill raises moral issues. Parental ease, embracing oneself, and criteria for permission and assent. Inadequate comprehension of local regulations regarding medical devices (e.g., IRB). It might not be feasible or desirable for researchers to employ pseudo devices and masking in studies. No legally binding guidelines exist for the age branding of medical devices. FDA reviewers' distinctive exposure to pediatric devices
Financial considerations	Inadequate facilities and finance for layout, assessment, and implementation. Reimbursement rates for adulthood are reduced and their tactics are more intricate

## Conclusions

An extensive study was undertaken to determine the policies in Japan and the US that promote the manufacturing of pediatric medical devices. Our investigation concluded that the efforts of the two nations are identical, including waivers of regulatory clearance, prioritized review, and subsidies for application expenses and R&D costs. The establishment of pediatric medical devices involves accelerating regulatory body clearance through scrutiny, fostering international development, and setting forth alternative protocols for rigorous clinical trials to ensure the device's safety and effectiveness. Prospective research on the establishment of clinically relevant, device-specific in vitro test methods may facilitate rapid innovation and regulatory clearance of medical devices, notably pediatric devices. There is still optimism that this research area can help advance the development of reliable and safe pediatric medical devices.

Our involvement with the "HBD-for-Children program" demonstrated the crucial importance of collaboration among academic institutions, businesses, and government agencies to promote the early development of pediatric medical devices. Pediatric medical devices are vital for children's health, but their development and commercialization are complex and often misunderstood. Regulatory, technical, physiological, and financial hurdles widen the gap between pediatric and adult innovation. While progress has been made, broad cooperation and prioritized funding from stakeholders, Congress, and the FDA are necessary to narrow this gap. Legislative and regulatory mechanisms can drive change in the heavily regulated medical device market.
